# Lack of emotional gaze preferences using eye-tracking in remitted bipolar I disorder

**DOI:** 10.1186/s40345-018-0123-y

**Published:** 2018-07-03

**Authors:** John R. Purcell, Monika Lohani, Christie Musket, Aleena C. Hay, Derek M. Isaacowitz, June Gruber

**Affiliations:** 1 Department of Psychological and Brain Sciences, Indiana University, 1101 E. 10th St., Bloomington, IN 47405 USA; 2 Department of Psychology, University of Utah, 201 Presidents Circle, Salt Lake City, UT 84112 USA; 3 Department of Psychology, University of Pittsburgh, 210 South Bouquet St, #4209, Pittsburgh, PA 15260 USA; 4 Department of Psychological and Brain Sciences, Boston University, 648 Beacon St., 6th Floor, Boston, MA 02215 USA; 5 Department of Psychology, Northeastern University, 360 Huntington Ave., Boston, MA 02115 USA; 6 Department of Psychology and Neuroscience, University of Colorado Boulder, 345 UCB, Muenzinger D321C, Boulder, CO 80309 USA

**Keywords:** Emotion, Attention, Eye-tracking, Bipolar disorder

## Abstract

**Background:**

Bipolar disorder is associated with heightened and persistent positive emotion (Gruber in Curr Dir Psychol Sci 20:217–221, 2011; Johnson in Clin Psychol Rev 25:241–262, 2005). Yet little is known about information processing biases that may influence these patterns of emotion responding.

**Methods:**

The current study adopted eye-tracking methodology as a continuous measure of sustained overt attention to monitor gaze preferences during passive viewing of positive, negative, and neutral standardized photo stimuli among remitted bipolar adults and healthy controls. Percentage fixation durations were recorded for predetermined areas of interest across the entire image presentation, and exploratory analyses were conducted to examine early versus late temporal phases of image processing.

**Results:**

Results suggest that the bipolar and healthy control groups did not differ in patterns of attention bias.

**Conclusions:**

Findings provide insight into apparently intact attention processing despite disrupted emotional responding in bipolar disorder.

## Background

Bipolar disorder (BD) is a severe and chronic psychiatric condition associated with functional impairment and disability (Fagiolini et al. 2005; Michalak et al. 2007). Recent models of BD stress the importance of difficulties in positive emotion processing (Gruber 2011; Phillips and Vieta 2007). An important next step is to characterize processes that may underlie and contribute to these patterns of emotion disturbance. One promising route is to explore attentional biases, which have been shown to play an important role in depression (e.g., Gotlib et al. 2004; Joormann et al. 2007). However, comparably less is known about how attentional biases contribute to disturbances in positive emotion characteristic of BD.

### Emotion disturbance in BD

Recent models suggest that individuals with BD experience heightened and persistent elevations in positive emotionality (e.g., Gruber 2011). This pattern is consistent with psychosocial models of BD that implicate heightened reward seeking and goal-striving (e.g., Alloy et al. 2009; Johnson 2005). For example, individuals at risk for BD and remitted BD adults self-report greater positive affect than healthy controls in response to emotional films (Gruber et al. 2011a, b), photos (M’Bailara et al. 2009), and at the prospect of earning rewards (Meyer et al. 2001). BD individuals and individuals at risk for BD also exhibit increased parasympathetic reactivity in response to emotional stimuli such as films, photos, and memories (Gruber et al. 2008, 2009; Sutton and Johnson 2002). Neuroimaging studies reveal that people with BD exhibit increased activity in brain regions typically associated with reward (e.g., ventral striatum) to positive stimuli (e.g., Dutra et al. 2015; Wessa et al. 2007). Heightened positive emotionality differentiates BD from major depressive disorder (Gruber et al. 2011a), and has important implications for psychosocial treatments aimed at reducing positive emotionality and subsequent manic episodes (e.g., Johnson 2005).

Abnormalities in negative emotionality might also be expected in BD, given the characteristic frequent and recurrent episodes of depression (Judd et al. 2003). However, the current literature suggests that people diagnosed with and at risk for BD may not differ from healthy controls in their emotional responses to negative stimuli including negative social feedback (e.g., Ruggero and Johnson 2006) and interpersonal criticism (Cuellar et al. 2009). At the same time, individuals with BD do report increased tendencies toward behavioral inhibition and neuroticism which is associated with increased negative affect (e.g., Alloy et al. 2008; Meyer et al. 2001; Murray et al. 2007).

### Coinciding cognitive and emotional processes

Though many studies have evaluated abnormal emotional reactivity in a variety of populations, a critical next step includes isolating the correspondingly impaired cognitive processes in BD (Gruber 2011). Cognitive processes such as attention have long been recognized as contributing to, and being affected by, emotion (e.g. Schwarz 2000; Shimojo et al. 2003), and visual attention in particular has been shown to impact emotional responding (e.g., Cacioppo et al. 2000). One methodology well-suited to examine visual attention for emotional events is eye-tracking technology, which has allowed researchers to elucidate different patterns of visual attention to negative stimuli in mood disorders such as depression and BD (Gotlib and Joormann 2010; Gotlib et al. 2004; Mathews and MacLeod 2005).

Research suggests that adults diagnosed with depression take longer to disengage attention away from sad faces, which has been predictive of sustained negative mood (Kellough et al. 2008; Sanchez et al. 2013). A meta-analytic review of eye-tracking studies has also found that failing to attend to positive events was associated with reductions in pleasure in depressed individuals (Armstrong and Olatunji 2012). Research on BD has found impaired recognition of negative facial expressions, failure to demonstrate attentional biases towards negative stimuli (Elliott et al. 2000; Lembke and Ketter 2002), and difficulties maintaining negative emotional information (Gruber et al. 2013). These findings suggest that BD may be associated with attention *away* from negative stimuli, while depression is associated with attention *toward* it.

There has been comparatively less work examining attentional biases underlying *positive* emotion. Wadlinger and Isaacowitz (2008) found that after experimentally training healthy adult participants to selectively attend to positive information, they spent significantly less time looking at negative stimuli following the attentional training. Similarly, trait happiness is associated with increased attentional bias toward a variety of positive stimuli (Raila et al. 2015), and optimism is associated with gaze preferences away from negative health-related images (e.g., cancer tumor images; Isaacowitz 2005). Furthermore, older adults, who report higher and more stable levels of positive emotional states relative to younger adults (for review, see Lohani et al. 2013), display attentional preferences toward positive and away from negative faces (Isaacowitz et al. 2006b, 2008) and negative images (Noh et al. 2011).

However, studies on visual attention to emotional stimuli in BD have suggested that biases may be mood-congruent. For example, research has found that mildly depressed BD participants demonstrate bias towards negative and away from positive words using a modified dot-probe task compared with controls (Jongen et al. 2007). Other studies have utilized “free-looking” tasks to simultaneously display several images to subjects have demonstrated that currently manic BD adults attend more to positive images, and currently depressed BD adults attend less to positive images, as compared to controls (García-Blanco et al. 2013, 2014, 2015, 2017; Leyman et al. 2009).

These recent studies have suggested that selective attention may coincide with positive and negative mood states in BD, and that these attentional biases are not present during periods of remission. This theory has been bolstered by studies that report no group differences between those with remitted BD and healthy controls in attention bias for positive or negative faces using emotional dot-probe, and free-view, tasks (e.g., Peckham et al. 2015, 2016). Thus far, a substantial body of literature suggests that attentional processes may not harmoniously fit into the model of BD as a disorder of positive emotionality throughout all mood phases of the disorder. However, findings thus far have only investigated attentional biases to differently-valenced, simultaneously presented affective stimuli.

### The present investigation

The present investigation aimed to explore attentional biases toward emotionally relevant stimuli in BD by employing continuous eye-tracking during individually presented images. By presenting images one at a time, the current study constrained participants’ potential attentional biases to affectively salient vs. non-salient areas, mimicking visual field processing in a more ecologically valid way. Using this methodology, individual and age-group differences in attentional biases have been found within non-clinical populations (Isaacowitz and Choi 2011; Wadlinger and Isaacowitz 2008). For instance, compared to younger adults, older adults have been found to focus their visual attention away from more negative regions and towards non-affective regions of an image (Noh et al. 2011). Using this method, we investigated whether individuals with remitted BD would deploy attention toward more salient negative or positive regions, as opposed to the non-emotional regions, of images, and whether or not these patterns of gaze fixation would differ from healthy controls. Considering the positive emotion persistence observed in remitted BD, two hypotheses were formed (Gruber 2011). The first predicted that individuals with BD would demonstrate specific attention biases towards positive emotional stimuli (i.e. *positive amplification)*. The other non-mutually exclusive hypothesis predicted that individuals with BD would demonstrate specific attentional biases away from negative emotional stimuli (i.e. *negative attenuation*).’

We also conducted post hoc exploratory analyses to investigate early, relative to late, phases of image viewing as well as sustained attention across the entire duration of image viewing. This approach has shown promise in other domains of emotion and attention processing such that individuals who report higher trait levels of happiness have shown increased dwell time and fixation counts to positive compared to neutral stimuli in later, rather than early, stages of visual attention (e.g., Nakayama and Mackeben 1989; Raila et al. 2015).

## Methods

### Participants

Participants were recruited as part of a larger study using posted flyers, online advertisements (e.g., http://www.craigslist.org), and outpatient mental health referrals. Participant sample size was based on common standards in research with severe psychiatric samples at the time of recruitment (i.e., between 2012 and 2013) and included 35 individuals diagnosed with BD type I, currently remitted, and a healthy control (CTL) group comprising 32 individuals who did not meet current or lifetime criteria for any DSM-IV-TR Axis I disorder (First et al. 2007). BD participants were currently remitted (i.e., not in a current manic depressed, or mixed mood phase in the past month) to examine patterns of attention-related biases independent of current mood phase. Participants in the BD group were not excluded on the basis of comorbid Axis I disorders (aside from substance or alcohol abuse/dependence in the past 6 months) to ensure a more ecologically valid sample (e.g. Kessler et al. 2005). Exclusion criteria for all participants included head trauma, cognitive impairment, stroke, neurological disease, severe medical illness, or current alcohol or substance abuse/dependence in the past 6 months. All participants had normal or corrected vision by glasses or soft contact lenses. Additional tasks not relevant to the present study were also conducted the same day.^1^

### Measures of clinical functioning, cognitive functioning, and visual acuity

#### Diagnostic evaluation and assessment of global functioning

Diagnoses were determined using the Structured Clinical Interview for DSM-IV (SCID-IV; First et al. 2007). As published in Hay et al. (2015), diagnostic ratings between the SCID interviewer matched an independent rater for a subset of participants from the larger study protocol.^2^ The Global Assessment of Functioning (GAF; Luborsky 1962) Scale was used to assess current general functioning on a scale from 1 (lowest level of functioning) to 100 (highest level of functioning). Additional information about illness duration, age of onset, and lifetime number of mood episodes was also collected.

#### Mood symptoms

Clinician-rated symptoms of mania were assessed using the Young Mania Rating Scale (YMRS; Young et al. 1978) and current depression symptoms using the Inventory of Depressive Symptomatology (IDS-C; Rush et al. 1996). See Hay et al. (2015) for inter-rater reliability for the YMRS and IDS-C. Current remitted mood status was verified according to SCID-IV criteria and cutoff scores on the YMRS (≤ 7), and IDS-C (≤ 11), which reflected symptomatology within the last 7 days.

#### Working memory and mental status

Participants were administered the letter-number sequencing subtest of the Wechsler Adult Intelligence Scale-IV (WAIS-IV; Pearson 2008). Raw scores (ranging from 0 to 21) were calculated as the total number of trials correct, from which WAIS-IV age-normed scaled scores were computed. The Mini-Mental State Examination (MMSE) was used as a brief, objective measure of cognitive capability (Folstein et al. 1975). Raw scores (ranging from 0 to 30) were calculated as the total number of trials correct. Threshold eligibility was set at ≥ 24, and all participants met or exceeded this score.

#### Visual acuity

Visual acuity was measured using the Snellen Eye Chart Test (Snellen 1862) that tested participants’ ability to distinguish small details by identifying different optotype letters. Participants were positioned 20 feet away from the Snellen chart in a well-lit room. Each score was given based upon a participant’s ability to accurately read an entire line of the smallest distinguishable letters using the left eye (i.e. after covering the right eye).

### Emotional stimuli

**Fig. 1:**
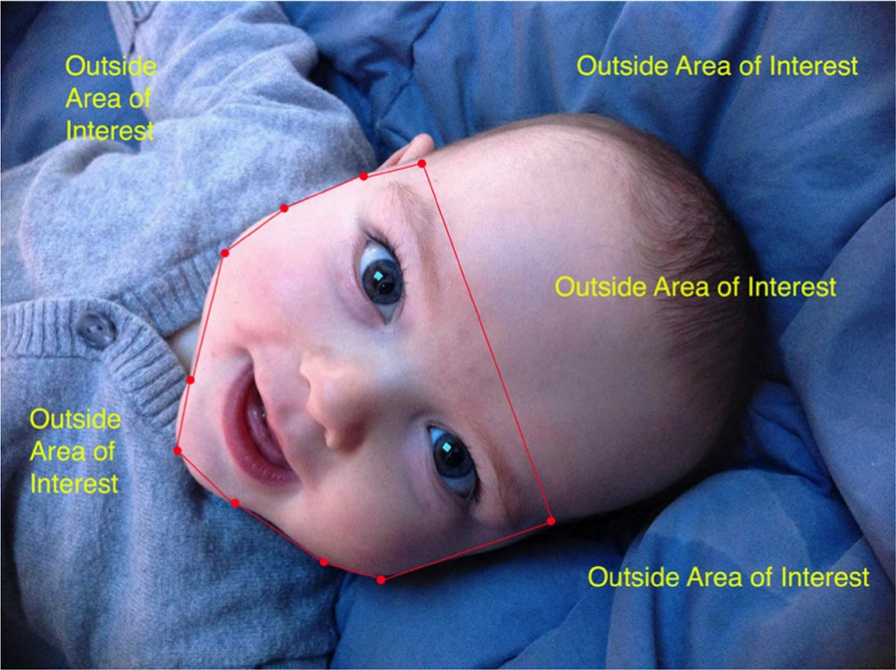
Example area of interest (AOI). The area within the red lines would be coded as a positively valenced AOI and all surrounding content would be coded as outside of the AOI. No boundaries (i.e. red lines) identified the AOIs for participants during experimentation

Emotional stimuli included 84 images from the International Affective Picture System classified according to valence intensity, and matched on normative arousal ratings across the 28 positive (*M* = 5.66, *SD* = 0.83) and 28 negative (*M* = 5.00, *SD* = 0.98) images (Lang et al. 1999). Less ideally, the 28 neutral images were less arousing on average (*M* = 3.15, *SD* = 0.53), but this is typical for research utilizing neutral IAPS stimuli (Schneider et al. 2016). For the present investigation, the affectively salient areas of each image were determined by tracing areas of interest (AOIs; e.g. Fig. 1) using a coding procedure similar to that of previous studies (e.g., Wadlinger and Isaacowitz 2008; Noh et al. 2011).

### Emotion-related gaze task

An Applied Science Laboratories eye tracker, Model 504 (Bedford, MA) recorded the movements and position of participants’ left eye sixty times per second with a camera and a non-invasive beam of infrared light. The visual fixations were defined as those series of gazes in which an individual stayed within 1° visual angle for 100 ms or longer (Manor and Gordon 2003). Visual fixations were recorded throughout the presentation of each image on a 15 × 12″ Dell desktop computer and calculated using GazeTracker software (Eye Response Technologies, Inc., Charlottesville, VA). To eliminate possible order effects, participants viewed all 84 images in one of three randomly generated orders, and were given the following instructions: “*Watch each image naturally, as you would if at home while watching TV. You can look anywhere on the screen, but try not to turn your head away from the screen.”* Each trial consisted of a single image, displayed at a size of 12 × 10.25 in. (5.0 s), followed by a buffer slide with a fixation cross (0.5 s) to realign gaze to the center of the screen.

### Procedure

After obtaining informed consent, trained interviewers administered the diagnostic, symptom and cognitive measures. Next, participants’ left eye visual acuity was tested. In an individual testing room, participants sat approximately 28″ away from a computer monitor. The height of the seat was also adjusted so that the participants’ gaze would naturally fall in the middle of the screen while keeping their heads in a neutral position. Participants were oriented to the task both verbally by the experimenter and through a short slide presentation. The eye-tracker was then calibrated to each participant’s left eye, using 17 points displayed in two concentric squares on the screen, ensuring that the tracker accurately recorded within 1° visual angle of each point (Isaacowitz et al. 2006a, b). Participants then viewed a series of images during the emotion-related gaze task. The entire task (instruction, calibration, and experimental trials) took approximately 30 min. Additional tasks unrelated to the eye-tracking task were also completed during this experimental session that same day. At the end, participants were debriefed and compensated.

### Data analyses plan

The goal of this project was to compare differences in visual attention within and outside of emotional AOIs in the CTL versus BD group. First, we examined group differences in the average percent time participants fixated on emotional AOIs across the *entire* image presentation (5.0 s) to understand overall attentional biases for different image valences. Second, we examined group differences in early (0.0–2.5 s) and late (2.51–5.0 s) phases of image presentation separately (by examining the average percent time fixated on *emotional* AOI in late − early phases) to understand group differences in attentional focus towards images in the later phase of image viewing after accounting for fixation duration in the early phase. This analysis was exploratory in nature, and implemented post hoc after discovering that visual attention in later stages (i.e. 10–15 s compared to 1–10 s) of visual attention to positive stimuli has been positively associated with trait levels of happiness within non-clinical subjects (Raila et al. 2015). Whenever assumptions of sphericity were not met, adjusted degrees of freedom are reported in the results using Huynh–Feldt estimates of sphericity.

## Results

### Participant characteristics

Following convention in previous eye-tracking studies (e.g., Isaacowitz and Choi 2011), participants were excluded from the final analysis for incomplete data due to track ability issues including problematic pupils, glare from eyeglasses (10 BD, 5 CTL participants) and/or other technical problems experienced during their sessions (1 BD, 2 CTL participants). This left a remaining sample of 24 BD and 25 CTL participants available for final data analysis. These participants were successfully tracked for at least 65% of each eye-tracking session (e.g., Isaacowitz and Choi 2011), for an average of 91.20% successful tracking overall. Trackable and nontrackable participants within each group did not differ on demographic, cognitive, or visual measures (*p*s > 0.05), and groups did not differ in percentage of trackable data (92.16 and 90.15% overall; *F*(1,47) = 0.92, *p* = 0.34, η^2^ = 0.001).

As seen in Table 1, BD and CTL participants did not significantly differ with respect to age, gender, ethnicity, or education (*p*s > 0.05). Not surprisingly, the BD group scored lower on global functioning than the CTL group. Although all groups scored below YMRS (≤ 7) and IDS-C (≤ 11) cutoffs, BD participants scored higher than CTL participants on the IDS-C and YMRS, and thus symptoms were controlled for in the main analyses. The groups did not differ on the baseline cognitive measures (*p* > 0.05). There were no main effects for order (*ps* > 0.13) or gender (*ps* > 0.12) for all main variables. Finally, BD participants were currently remitted for an average of 27.57 months (*SD* = 31.99).

**Table 1:** Demographic, clinical and visual acuity participant characteristics

	BD (*n* = 24)	Control (*n* = 25)	Statistic
Demographic			
Age (years)	33.88 (12.82)	32.16 (9.59)	*F* = 0.29
Female (%)	58.33%	52%	*χ*^*2*^ = 0.20
Caucasian (%)	87.50%	92%	*χ*^*2*^ = 2.71
Education (years)	14.88 (1.94)	15.86 (2.38)	*F* = 2.41
Employed (%)	58.33%	64%	*χ*^*2*^ = 1.66
Partnered (%)	41.66%	60%	*χ*^*2*^ = 1.64
Number children	0.54 (0.98)	0.32 (0.80)	*F* = 0.76
Annual income			*χ*^*2*^ = 11.78
< $10K	8 (33%)	3 (12%)	
$10K–$25K	10 (42%)	5 (20%)	
$26K–$50K	2 (8%)	5 (20%)	
$51K–$75K	1 (4%)	6 (24%)	
$76K–$100K	0	3 (12%)	
> $100K	3 (13%)	3 (12%)	
Cognitive			
Working memory	11.46 (2.73)	12.16 (3.39)	*F* = 0.63
MMSE	28.13 (1.92)	28.88 (1.33)	*F* = 2.57
Clinical			
YMRS	1.79 (2.06)	0.48 (0.71)	*F* = 8.99*
IDS-C	4.25 (2.75)	2.12 (2.49)	*F* = 8.08*
GAF	70.94 (8.33)	88.56 (4.78)	*F* = 83.121
Age at onset (years)	16.42 (4.55)	–	–
Illness duration (years)	17.46 (13.57)	–	–
# Depressive episodes	22.48 (30.27)	–	–
# Manic episodes	15.65 (24.33)	–	–
Visual acuity			*χ*^*2*^ = 2.78
20/13	2 (8.3%)	2 (8%)	
20/15	1 (4.2%)	2 (8%)	
20/20	6 (25%)	9 (36%)	
20/25	6 (25%)	5 (20%)	
20/30	5 (20.8%)	4 (16%)	
20/40	4 (16.7%)	2 (8%)	
20/50	0 (0%)	1 (4%)	

*BD* bipolar I disorder group, *Employed* employed full-time or part-time; *Partnered* married or live-in-partner, *YMRS* Young Mania Rating Scale, *IDS-C* inventory to diagnose depression, *GAF* global assessment of functioning; *Age at Onset* age of first depressive or manic episode; *# Comorbid disorders* the number of current DSM-IV-TR axis I comorbidities. *# Medications* the number of psychotropic medications currently taken (including anticonvulsants, lithium, neuroleptics, anxiolytics, stimulants, antidepressants, and sedative-hypnotics). Mean values are displayed with standard deviations in parentheses where applicable

* *p* < 0.05 for BD and CTL

### Validation of emotional stimuli

To ensure the validity of our emotional stimuli 100 independent judges (54.0% Female, 49.0% Caucasian, mean age = 33.69 years (*SD* = 9.75), recruited via Amazon Mechanical Turk (Buhrmester et al. 2011; Gosling et al. 2004), rated both the valence and arousal of each individual AOI, and surrounding non-AOI content, on 9-point, bipolar scales (e.g. *extremely unpleasant to extremely pleasant*). AOIs within positive images were rated as significantly more pleasant (*M* = 6.34, *SD* = 0.67) and arousing (*M* = 5.02, *SD* = 0.91) than the rest of image area (valence: *M* = 5.96, *SD* = 0.71; arousal: *M* = 4.63, *SD* = 0.86), *t*(99) = 8.85, *p* < 0.001 for valence and *t*(99) = 6.14, *p* < 0.001 for arousal. Similarly, AOIs within negative images (*M* = 2.59, *SD* = 0.40) were rated as significantly less pleasant (*M* = 3.02, *SD* = 0.92) and more arousing (*M* = 5.90, *SD* = 1.07) than the rest of the image areas (valence: *M* = 3.90, *SD* = 0.98 arousal: *M* = 5.27, *SD* = 1.02), *t*(99) = − 11.47, *p* < 0.001 for valence and *t*(99) = 8.234, *p* < 0.001 for arousal.

### Main analyses

In order to examine the differences in sustained attention between the BD and CTL groups we conducted a 2 (Group: BD, CTL) × 3 (Valence: Positive, Negative, Neutral) repeated-measures ANCOVA for the average percent time participants fixated on emotional AOI across the entire image presentation (5.0 s), while controlling for depression and manic symptom levels (YMRS and IDS-C scores).^3^ Mauchly’s test indicated that the assumption of sphericity had been violated (*p* < 0.05); therefore, degrees of freedom were corrected using Huynh–Feldt estimates of sphericity. Results revealed a main effect of Valence, *F*(1.73, 77.84) = 54.35, *p* < 0.001, η^2^ = 0.55, with more percent time fixated on Neutral images (*M* = 70.52, *SE* = 1.14), followed by Positive images (*M* = 67.85, *SE* = 1.20), and Negative images (*M* = 55.76, *SE* = 1.62), thus not supporting the positive amplification nor negative attenuation hypotheses. The Group main effect (*p* = 0.50) and Group x Valence interaction were not significant (*p* = 0.36). Pairwise comparisons for each Valence type were conducted to compare the BD versus the CTL group but were not significant, (*p*s > 0.21). No significant differences were found in fixation duration patterns for the entire image presentation time.

### Exploratory analyses

Next, we conducted exploratory analyses to explore whether attentional profiles were sensitive to the temporal dynamics of visual attention by examining the average percent time participants fixated at *emotional* AOI separately for the early and late phases of image presentation by computing a difference score by subtracting fixation % from the late − early phase scores (Table 2). There was a marginally significant effect of valence, *F*(2, 90) = 2.95, *p* = 0.06, η^2^ = 0.06. No main effect of group (*p* = 0.79) or group × valence interaction effect was found, (*p* = 0.43). None of the between-subjects comparisons were found significant, *p*s > 0.34.^4^

**Table 2:** Mean percent fixation in non-emotional AOI regions in the early and late phases of image presentation for each picture type

Phase	Image-type	Control	BD
Early	Positive images	0.49 (0.03)	0.44 (0.04)
Late	Positive images	0.54 (0.03)	0.50 (0.004)
Early	Negative images	0.63 (0.04)	0.58 (0.05)
Late	Negative images	0.67 (0.04)	0.69 (0.06)
Early	Neutral images	0.45 (0.02)	0.41 (0.03)
Late	Neutral images	0.46 (0.03)	0.44 (0.05)

Mean values are displayed with standard error values in parentheses where applicable

*BD* bipolar I disorder group

## Discussion

Bipolar disorder (BD) is a chronic psychiatric disorder that is associated with heightened and persistent positive emotion and reward sensitivity, even during periods of remission from mood episodes (M’Bailara et al. 2009; Gruber 2011; Johnson 2005). Attentional biases toward positive emotion stimuli have been shown to be related to trait positive emotionality in multiple populations, and BD individuals currently experiencing manic symptoms have been shown to show attentional biases to positive visual stimuli; however, recent literature suggests that these biases may not present in *remitted* BD (García-Blanco et al. 2013, 2014, 2015, 2017). The current study sought to clarify the potential attentional biases to emotionally-valenced stimuli in remitted BD patients, as compared to healthy controls, using a continuous eye-tracking methodology (which is novel in this population). Overall, we found no significant group differences in fixation time towards emotionally-valenced stimuli, suggesting an area of relative strength in remitted BD patients.

### No support for positive amplification or negative attenuation perspectives

We examined whether remitted BD was associated with attentional bias towards positive stimuli or away from negative stimuli, both of which may facilitate sustained positive mood perhaps even independent of current mood state. Somewhat surprisingly, we did not find support for either perspective. The absence of support for the *positive amplification* hypothesis is consistent with previous findings suggesting that visual biases are mood-dependent in BD (Lembke and Ketter 2002), and do not differ from non-clinical controls during remission (García-Blanco et al. 2013, 2014, 2015, 2017; Peckham et al. 2015, 2016). However, these findings are somewhat inconsistent with previous research in which high trait hypomania (Putman et al. 2007) were associated with gaze preferences for positive stimuli.

The absence of support for the *negative attenuation* perspective is also surprising as those with BD have exhibited impaired recognition of negative facial expressions and failed to demonstrate attentional biases towards negative stimuli (Elliott et al. 2000; Lembke and Ketter 2002) and demonstrate difficulties maintaining negative emotional information (Gruber et al. 2013). However, this absence of group effects is consistent with other recent null group findings in remitted adults with BD across experimental dot-probe and free-viewing eye-tracking tasks (Peckham et al. 2015, 2016).

### Implications of findings for positive emotion disturbance in BD

These findings are not necessarily inconsistent with positive emotion persistence in BD, which is quantified by emotion responsivity and reactivity. Rather, they suggest that persistent emotional reactivity and responsiveness may be unrelated to bottom-up involuntary attention biases to positive emotion stimuli. However, more research is required to elucidate the relationship between emotion, attention, and symptomatology in BD (for review, see Lima et al. 2017).

## Conclusion

The current study expanded further upon the body of emotional visual biases in remitted BD by assessing fixation time within affective areas (i.e., AOIs) of an image. These findings broaden our understanding of emotion processing in BD by assessing whether visual attention is differentially allocated within a single emotional image, rather than between several emotionally valenced images. This distinction is important, as visual attentional tasks typically present simultaneous, distinct options for gaze, which may not mirror ecologically-valid visual scenes that include both emotionally salient regions as well as less emotionally salient regions. Overall, the results suggest that remitted BD is not characterized by differential visual attention biases to affective regions of stimuli compared to non-clinical controls.

## Limitations

Findings from the present study should be interpreted within the confines of several limitations. First, although the images used in the present study are standardized and reliable elicitors of emotions, it could be argued that the results may not be generalizable to everyday emotional experiences or visual scenes in the lives of BD patients. Thus, it will be important for future studies to assess attention biases using stimuli that are more ecologically valid (e.g., personalized emotional images), and perhaps more dynamic in nature (e.g., remembering the temporal sequence of an emotional event) and personally salient (e.g., autobiographical memories). Second, the use of complex static images may have introduced variance in emotional gaze responses, which may have driven observed attentional patterns towards or away from the image (e.g., Raila et al. 2015). Third, we acknowledge that our sample sizes were relatively modest despite mirroring sample sizes typically reported in experimental psychopathology research among BD patients (e.g., Robinson et al. 2006). Future studies would benefit from larger sample sizes. Fourth, the sample consisted largely of Caucasian participants and results may not generalize to a more diverse sample. Fifth, individuals in the BD group were not excluded on the basis of comorbidities to ensure a more ecologically valid sample so future research should examine how the presence of specific comorbid disorders influences emotional attention biases. Finally, given the possible confound of psychotropic medication, future paradigms with random assignment to different medication classes are warranted. Specifically, future research might include a control group that is matched on the same comorbid conditions, as well as assignment of BD individuals on different medication classes (e.g., antidepressants, mood stabilizers, anxiolytics).

## Future directions

While the current study was not designed to investigate emotion regulation, it sheds light on an important cognitive component of emotion regulation; namely attention allocation to affective stimuli or situations (Gross 2015). Difficulty with selecting and independently implementing appropriate positive emotion regulation strategies is characteristic of BD (Gruber et al. 2012; Hay et al. 2015). Despite these issues, visual attention to affective stimuli has not been associated with self-reported emotion regulation (Peckham et al. 2016). Our results indicate that BD is not inherently characterized by a universal attentional bias toward or away from affective stimuli; however, a more targeted evaluation of whether or not visual attention biases contribute to, or are impacted by, the implementation of emotion regulation strategies is necessary. If visual attention to affective stimuli is unaltered in individuals with BD during emotion regulation efforts, other components or stages of emotion regulation such as appraisal, reactivity, and strategy implementation could be targets of treatment and intervention to improve emotion regulation in individuals with BD.

## Footnotes

1 One other publication to date has arisen from the same broader protocol (e.g., Hay et al. 2015). The other tasks administered on the same day not yet published included experimental tasks measuring baseline emotion responding, attentional rubbernecking, and emotion and memory. We also administered a larger battery of questionnaires at the end of the study that included medication history, health information, suicidal history information, Care Satisfaction Questionnaire (e.g., Morris et al. 2005), Altman Self-Rating Mania Scale (Altman et al. 1997), BDI-SF (Beck and Beck 1972), STAI-state (Spielberger et al. 1970), SWLS (Diener et al. 1985), modified Differential Emotions Scale (Cohn et al. 2009), ERQ (Gross and John 2003), Response Style Questionnaire-Distraction subscale (Nolen-Hoeksema and Morrow 1991), Emotion Beliefs Survey (Tamir et al. 2007), Berkeley Expressivity Questionnaire (Gross and John 1997), Difficulty with Emotion Regulation Scale (Gratz and Roemer 2004), and a questionnaire assessing positive and negative affect for the Hay et al. (2015) study cited above.

2 We provide additional details for interested readers beyond the typically brief description of inter-rater procedures common in mood-disordered studies (Gruber and Weinstock 2018). Specifically, we note for this study no consensus meetings took place for clinical diagnosis and severity ratings, as the rater performed the ratings independently (i.e., did not look at the interviewer’s scores beforehand) and did not correct or modify her scores at any point. As is common practice, both the interviewers and rater consulted if diagnostic questions arose as needed. As such, the ratings reported above from Hay et al. (2015) reflect original (i.e., pre-meeting) values. Of note, this is distinct from other previously published studies from the same laboratory that do typically hold consensus meetings where major discrepancies (i.e., errors) are resolved and final ratings reflected post-meeting ratings (for a non-exaustive list of examples from our research team using this approach see: Dutra et al. 2015; Kang and Gruber 2013; Gruber et al. 2016; Ong et al. 2017). Both approaches with or without consensus meetings from this laboratory team frequently yield “almost perfect” (i.e., above 0.90) levels of agreement (e.g., McHugh 2012).

3 The same analyses were performed without controlling for symptomatology and results were still null.

4 Although not part of the main results reported in this study, for full transparency we note that we also examined the average percent time participants fixated at *non-emotional* AOI. We then computed a difference score by subtracting fixation % from the late − early phase scores. No significant effects of valence (*p* = 0.11), group (*p* = 0.21), or valence × group were significant (*p* = 0.21). A post hoc comparison of CTL versus BD group for negative images was significant: *t*(47) = 2.58, *p* = 0.01, Cohen’s d = 0.75 (large effect). Examination of the mean values for the Early and Late phases of image presentation appear to suggest that the BD group fixated significantly more at non-emotional regions in the Late phase (versus the early phase) of viewing negative images compared to the CTL group. Given the small sample size and large effect size (especially in the absence of other significant group differences; ps > 0.64), we believe this finding should be interpreted with extreme caution.
